# IMMUNOMODULATING EFFECTS OF THE PURIFIED HEV B 13 FRACTION ON SEPTIC RATS

**DOI:** 10.1590/0102-6720201700020004

**Published:** 2017

**Authors:** Maxley Martins ALVES, Lilhian Alves de ARAÚJO, Fátima MRUÉ, Clayson Moura GOMES, Milton Adriano Pelli de OLIVEIRA, Roberpaulo Anacleto NEVES, Nelson Jorge da SILVA-JÚNIOR, Paulo Roberto de MELO-REIS

**Affiliations:** 1 Laboratory of Experimental and Biotechnological Studies of the Post-Graduate Program in Environmental Sciences and Health, Area V, Campus I, Pontifical Catholic University of Goiás;; 2 Laboratory of Cytokines, Institute of Tropical Pathology and Public Health, Federal University of Goiás, Goiânia, GO, Brazil

**Keywords:** Sepsis, Hev b 13, Immunomodulation.

## Abstract

**Background:**

Sepsis is a potentially life-threatening complication of an infection that occurs when chemicals released into the bloodstream to fight the infection trigger inflammatory responses throughout the body, especially in the acute phase of the disease, producing excessive pro-inflammatory cytokines, leading to multiple organ injury and death. The Hev b 13 fraction has demonstrated biological activity capable of inducing IL-10 production and shrinking inflammatory disease lesions.

**Aim:**

To investigate the immunomodulating effects of the Hev b 13 fraction on septic rats.

**Methods:**

*Acinetobacter baumannii* was injected into the peritoneal cavity of the animals after sustaining a lesion in the pancreas, with the stomach as an entry point. After 10 h of infection, they were euthanized for blood and lung collection, followed by total and differential leukocyte count, determination of cytokine level and histopathological analysis.

**Results:**

Administering a single dose of the Hev b 13 fraction 2 h after sepsis induction significantly decreased total leukocyte count. Higher IL-10 and IL-4 and lower IL-6 production shrank the lung tissue lesions compared to the control groups.

**Conclusion:**

The Hev b 13 fraction exhibits an anti-inflammatory tendency, with potential for sepsis treatment.

## RESUMO

**Racional:**

Sepse se correlaciona com a ruptura do complexo equilíbrio entre os mediadores inflamatórios, que principalmente na fase aguda da doença, produz exacerbadamente citocinas pró-inflamatórias levando a lesão de múltiplos órgãos e morte. A fração Hev b 13 tem demonstrado atividade biológica capaz de induzir a produção de IL-10 e regredir lesões de doenças inflamatórias.

**Objetivo:**

Investigar os efeitos imunomoduladores da fração Hev b 13 em ratos com sepse.

**Métodos:**

Foi injetado *Acinetobacter baumannii* na cavidade peritoneal dos animais após lesão no pâncreas e estômago como porta de entrada. Após 10 h de infecção, foi realizada eutanásia para coleta de sangue e pulmões, em seguida, contagem total e diferencial de leucócitos, dosagem de citocinas e histopatologia para análise.

**Resultados:**

A administração de dose única da fração Hev b 13, 2 h após a indução de sepse, diminuiu significativamente a contagem total de leucócitos. Associado a maior produção de IL-10 e IL-4, e menor de IL-6, atenuou as lesões nos tecidos pulmonares em comparação com os grupos controles.

**Conclusão:**

A fração Hev b 13 apresenta tendência anti-inflamatória, com potencialidades no tratamento da sepse.

## INTRODUCTION

The definition of sepsis covers situations in which the systemic inflammatory response syndrome triggered by suspected or confirmed infection is established. From the clinical point of view, the presentation of sepsis correlates to the different possibilities of interaction between man and microorganisms, and may occur from different initial foci^17^.

Its clinical manifestations - such as fever, edema, hypercoagulation, tachypnea and peripheral hypotension - derive from the systemic release of inflammatory mediators, called cytokines, by the defense and endothelial cells, playing an important role acting as chemical messengers^2^ ^,^ ^3^. There are still those who characterize sepsis as the rupture of the complex balance between pro-inflammatory and anti-inflammatory cytokines^10^.

The imbalance between these mediators is strongly related to severity and mortality in sepsis, which mainly in the acute phase of the disease produces exacerbated pro-inflammatory cytokines contributing to target organ damage, leading to multiple organ failure and death. The association between high concentrations of proinflammatory cytokines IL-1, IL-6 and TNF in the acute phase of sepsis and severe organ dysfunctions has been studied and proven^5^ ^,^ ^10^ ^,^ ^13^.

There is no effective medication on the market to achieve the counterbalance of the acute inflammatory response to date. Several treatment protocols have been proposed in order to reduce the morbidity and mortality induced by the inflammatory response in sepsis; however, despite great advances, the incidence of deaths due to it is still high, and it is necessary to search for new therapeutic resources^1^ ^,^ ^17^.

Recent research using a protein derived from *Hevea brasiliensis* (rubber tree) natural latex, called Hev b 13, has demonstrated biological activity capable of inducing the production of interleukin 10 (IL-10) in vitro, stimulating an anti-inflammatory response^19^. In other experiments using animal models of ulcerative colitis as well as rheumatoid arthritis, treatment of animals with the Hev b 13 fraction demonstrated a significant regression of the lesions under study^20^.

In view of the immunomodulatory potential of the purified Hev b 13 fraction, the aim of this study was to investigate its effects in rats with experimentally induced sepsis.

## METHODS

### Obtaining Hev b13 and animals

The purified fraction Hev b 13, derived from the natural latex of *Hevea brasiliensis*, was kindly supplied by the Pele Nova Biotechnology Laboratory lot 1502-243.

*Rattus norvegicus albinus* adult male Wistar rats, presenting body weight between 200-300 g, were collected from the Central Vivarium of the Pontifical Catholic University of Goiás. The animals were housed in individual polypropylene cages with solid floors, lined with sterilized shavings, according to international standards and the Brazilian Society of Sciences in Laboratory Animals. The environment was maintained at an average temperature of 21° C, ventilation system, dark light cycle, species specific diet and water ad libitum. The experiment started after the approval of the Ethics Committee on Animal Use of PUC-GO protocol no. 002/2013.

### Multidrug-resistant Acinetobacter baumannii strains 

The strains used in the experiment were obtained from the Laboratory of Clinical Analyzes of the state of Goiás, Brazil, cultivated, isolated and diluted to 6x108 CFU/mm^3^, with viability and profile analysis in the Laboratory of Microbiology of the Institute Carlos chagas/Fiocruz, Rio de Janeiro, Brazil through the nephelometric scale of Mc Farland. Subsequently, they were submitted to culture and antibiogram to confirm resistance to antibiotics.

### Induction of sepsis

The animals were anesthetized in the anterior muscle of the right thigh, with ketamine hydrochloride (50 mg/kg) and xylazine (8 mg/kg). Subsequently, antisepsis of the surgical region was performed with chlorhexidine degermant and then submitted to median laparotomy with traction and transverse section of the gastric wall of 0.5 cm, dissection and section of the retrogastric expiratory pancreas with ligation of the extremities using silk thread 2.0 followed by a transverse section of the duodenum in its second portion. The synthesis was carried out with polyester thread 3.0 cylindrical needle, both gastric and duodenal and careful evaluation of gastro and enterorraphy, checking the transit patency and complete hemostasis. At this time, 1.0 ml of the solution containing *Acinetobacter baumannii* (6x108) was injected into the peritoneal cavity and, after suturing the abdominal wall in two planes with polyester and nylon 3.0 threads.

### Groups and treatments

The treatment was performed according to the randomization in the animals in the second postoperative hour at a dose of 0.5 mg/kg Hev b 13 or 1 ml of 0.9% physiological solution. The groups were distributed as follows: Normal Control - animals not exposed to any type of procedure or treatment; Surgical Control - animals submitted only to the operation without strain inoculation and treatment; Treatment group Hev b 13 - animals submitted to surgery, with inoculation of strain and treatment with Hev b 13; Treatment Group Saline Solution 0.9% - animals submitted to surgery, with inoculation of strain and treatment with physiological solution. Euthanasia was performed at the 10^th^ postoperative hour for collection of biological material. This time was determined in a pilot study in which a significant worsening in the clinical signs of sepsis was observed.

### Leukogram and plaquetogram

They were performed in the COBAS XE 2100D equipment of ROCHE, by the methodology of citometry and electrical impedance. For differential counting, smears of new blood samples and stained with Panótico (Laborclin - Paraná - Brazil) were made to identify the leukocytes through their morphological specificities under optical microscopy (Nikon, Model - Eclipse E - 400X magnification).

### Histopathological analysis

The right lung was fixed in 10% formaldehyde solution and subsequently included in paraffin. Each block was prepared and sectioned in a microtome at 5 μm thickness and then stained with H&E for light microscope viewing. The images were obtained through the digital camera coupled to the microscope, with a Pinnacle Studio AV/DV Deluxe capture card. The analysis of the histopathological lesions of the lungs were classified as: (-) absent; (+) mild; (++) moderate; (+++) severe in the categories neutrophil infiltration, interstitial edema, congestion, haemorrhage, hyaline membrane and necrosis^18^.

### Sample preparation and Elisa

Left lung fragments were collected, weighed on a precision scale, packed in eppendorfs with 200 μl of 1X saline-phosphate buffer and stored in the freezer at -80° C until dosing. The samples were thawed and added with 15 ml/mg of PBS 1X, 10% lysis solution and 1% protease inhibitor (Sigma), manually macerated, homogenized by the Politron apparatus and subsequently centrifuged. The samples were collected for the IL-10, IL -4, IL-6 and TNF, through the enzyme-linked immunosorbent assay (ELISA), according to the manufacturer’s instructions in the BD-OptEIATM Biosciences kits.

### Statistical analysis

The concentrations of cytokines contained in the samples were calculated from the standard curve obtained by serial dilution. For multiple comparison ANOVA (Analysis of Variance) was used followed by the Bonferroni test, with significance level p<0.05. The results were expressed in picograms or nanograms of cytokines/milligrams of tissue and the calculation performed by the program GraphPad Prism version 6.05, with significance level p<0.05.

## RESULTS

### Resistance profile of Acinetobacter baumannii strains 

The analysis of the antimicrobial resistance profile of *Acinetobacter baumannii* strains used in the induction of sepsis is shown in Table 1, being resistant to 14 different types of antibiotics and sensitive only to tetracycline and trimetropim.

**TABLE 1:** Antibiogram of Acinetobacter baumannii strains

Antibiotic	MIC*	Standard
Amicacine	>32	Resistant
Amipiciline/Sulbactam	>16/8	Resistant
Cefepime	>16	Resistant
Cefotaxime	>32	Resistant
Ceftadizime	>16	Resistant
Ceftriaxone	>32	Resistant
Ciprofloxacin	>2	Resistant
Gentamicine	>8	Resistant
Imipenem	>8	Resistant
Levofloxacin	>4	Resistant
Meropenem	>8	Resistant
Piperaciline/Tazobactan	>64	Resistant
Tetracicline	< 4	Sensitive
Tobramicine	>8	Resistant
Trimetropim/Sulfadiazine	<2/38	Sensitive

* Minimal inhibitory concentration

**TABLE 2:** Descriptive analysis of the laboratory tests of white series and platelets, between the Normal Control, Surgical Control, Treatment SF 0.9% and Treatment Hev b13

Parameters (103 μl)	Normal control	Surgical control	Control SS	Hev b 13
Plaquetogram				
Mean (±SD)	399666 (±124073.9)	510600 (±174513.3)	397000 (±114024.1)	479480 (±214806.8)
Variation	258000-489000	304000-751000	241000-522000	100400-634000
Total leukocytes				
Mean (±SD)	2983 (±104.08)	3940 (±432.8)	4416 (±1133.2)*	2748 (±633.3)
Variation	2900-3100	3560-4410	3340-5700	1930-3600
Neutrophils				
Mean (±SD)	1252 (±46.7)	2125 (±7.95)	1869 (±956.429)	1096.28 (±392.655)
Variation	1209-1302	1282-2808	939.3-2957.4	579-1530
Lymphocytes				
Mean (±SD)	1125 (±75.18)	1794 (±666.2)	1797.3 (±103.109)	1171.4 (±322.097)
Variation	1064-1209	1118-2450	1666.5-1938	663-1453.5
Eosinophils				
Mean (±SD)	19.3 (±16.7)	11.6 (±20.2)	22.6 (±30.894)	37.56 (±15.013)
Variation	0-29	0-35	0-57	25-62
Monocytes				
Mean (±SD)	527 (±41.4)	527 (±151.7)	598.04 (±168.9)	429.32 (±287.951)
Variation	383-465	357-648	424.2-855	250.9-936
Basophils				
Mean (±SD)	49.6 (±18.0)	26.3 (±23.2)	22.56 (±30.894)	13.42 (±18.458)
Variation	0-62	0-44	0-57	0-36

SD=standard deviation; SS=saline *=significant difference (p<0.05) between the Normal and Hev b 13 groups compared with the ANOVA test followed by Bonferroni

### Laboratory analysis of leukocytes and platelets

In the comparison between the means of the laboratory tests of white series and platelets, the total leukocyte count was significantly lower (p<0.05) in the group treated with Hev b 13 (2748±633.3) in relation to the group treated with solution (4416±1133.2). The normal control group (2983±104.08) was also significantly lower than the saline group. For the other cellular specificities, there was no significant difference.

### Histological analysis of the lungs

**FIGURE 1-Effects:**
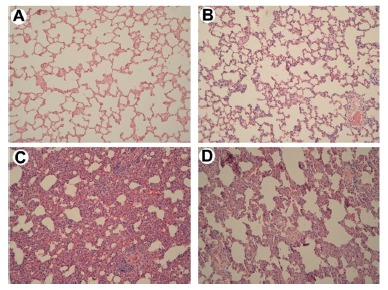
of Hev b 13 treatment on pulmonary histology of H&E (magnification 40X): A) pulmonary tissue from the Normal Control group without morphological changes; B) Surgical control group with slight thickening of alveolar septa and rare hemorrhagic spots; C) Saline solution group with marked thickening of alveolar septa, hemorrhage and inflammatory infiltrates; D) Hev b 13 group with moderate thickening of alveolar septa, mild hemorrhage and inflammatory infiltrates.

In the normal control group there were no morphological changes and were used for reference images (Figure 1A). In the surgical control there was mild edema of alveolar septa and rare hemorrhagic spots (Figure 1B). Acute thickening of alveolar septa with intra-alveolar hemorrhage, inflammatory infiltrates, and cell hyperplasia was observed in the saline treated group (Figure 1C), whereas interstitial inflammation in the Hev b 13 treated group was less severe, with moderate thickening of septa, mild hemorrhage and less inflammatory infiltrates (Figure 1D).

### Analysis of cytokines in lung tissue

**FIGURE 2:**
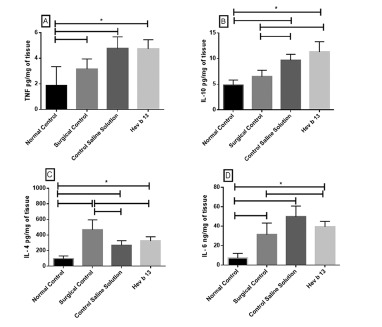
Effects of Hev b 13 on cytokine production in lung tissue from sepsis rats: after 10 h of subcutaneous treatment with 0.5 mg/kg Hev 13, the animals were euthanized, lung tissue samples were collected, including control, for cytokine dosage with n=8 individuals per group. Concentration values of IL-10 (2A), TNF (2B), IL-4 (2C) and IL-6 (2D) are expressed in picograms or nanograms of cytokines/milligrams of tissue. The horizontal line represents the mean±SD; *p <0.05 indicates the significant groups analyzed by the Anova test followed by Bonferroni.

TNF production in the group treated with Hev b 13 was similar to the group treated with saline solution and surgical group, with significant difference (p<0.05) only in relation to the normal group (Figure 2A).

Levels in the production of IL-10 in the group treated with Hev b 13 were increased with respect to all other groups, however significant (p<0.05) only between the normal and surgical groups (Figure 2B).

Regarding IL-4 levels, the group treated with Hev b 13 presented higher production than the group treated with saline; however, there was no significant difference between them. Interestingly, surgical control had significantly higher mean (p<0.05) in IL-4 production than in the other groups (Figure 2C).

In Figure 2D, IL-6 production in the group of Hev b 13-treated animals was lower than in the group treated with saline alone, but not significant. Surgical control had significant lower levels (p<0.05) between the saline treated group and increased compared to the normal group.

## DISCUSSION

The Hev b 13 fraction is an allergenic esterase, obtained from the natural latex of *Hevea brasiliensis* ^2^, which in highly purified fractions stimulated human monocytes in previous studies to produce elevated levels of IL-10 and reduce TNF, opening the way for the investigation of immunological reaction effects mediated by this fraction^19^ ^,^ ^20^.

Sepsis is a severe form of systemic inflammatory reaction, usually caused by bacterial infections leading to an uncontrolled increase in the inflammatory response^7^. In this sense, the Hev b 13 fraction was administered in septic rats and its immunomodulatory effects evaluated in this study.

To induce septic process in rats, stomach and pancreas are damaged serving as the gateway for the injection of *Acinetobacter baumannii*. This model was used to simulate everyday situations in Brazilian intensive care units, where this bacterium with resistance-building abilities compromises the survival of patients who are on intense acute inflammatory response, usually used as a site of surgical wound infection^6^ ^,^ ^11^.

In this study, total leukocytes from the Hev b 13 group showed decreased levels compared to the saline treated group. One hypothesis for this first result would be the protein redistribution of the leukocytes from the vascular compartment to the site of the injury. Therefore, the lungs were collected for histopathological analysis and dosage of inflammatory mediators, seeking possible tissue damages. Histopathological changes were found in the pulmonary tissues of septic animals. This occurrence coincides with other authors, since the lungs are the most frequently affected organs in severe sepsis^10^ ^,^ ^21^. However, alveolar destruction was surprisingly attenuated in animals receiving Hev b 13 treatment (Figure 1D), for animals receiving saline treatment alone (Figure 1C). This protection probably occurred due to inhibition of inflammation or oxidative stress, which are the two most important mechanisms responsible for organ damage in sepsis^12^, a fact that was consolidated after the results of the concentration of inflammatory mediators in lung tissues.

It was found that in animals treated with Hev b 13, the concentration of IL-6 in lung tissues, although not significant, was lower compared to the group treated with saline alone. Previous studies have shown that TNF and IL-6 are the cytokines most strongly associated with sepsis. Excess production of these inflammatory mediators induces endothelial and epithelial damage, vascular extravasation, edema and vasodilatation, favoring the development of multiple organ dysfunction syndrome^23^.

The decrease in IL-6 production in the lung tissue itself in this study may have attenuated the inflammatory process, consequently alveolar thickening. Our results are similar to the ones published by Teixeira et al.^19^, where the administration of Hev b 13 in mice with experimental colitis promoted reduction of inflammatory activity and leukocyte infiltration in the histological analysis of the distal colon. In another study, experimental arthritis was induced in mice and treated with the same fraction. The authors described a remarkable improvement in the histopathological findings of the knee joints, with a decrease in inflammatory activity^20^.

It was also observed that there was a greater trend in the production of the two anti-inflammatory cytokines dosed in this study, IL-4 and IL-10, in Hev b 13 treated animals compared to the saline-treated control group. Some studies have correlated the protective effects of IL-10 on sepsis with factors such as: septic process induction methodology (LPS-lipopolysaccharide or LPC-ligation and cecal puncture) and time of intervention^8^. But in general, the treatment of septic mice with IL-10 delays the onset of lethality, increases survival, and extends the therapeutic window^15^. In contrast, IL-10 administered after the development of severe septic shock, regardless of induction methodology, may have limited therapeutic benefit^9^.

 With regard to IL-4, it is known to play an important role in the pathogenesis of sepsis, but its precise function during the course of the disease remains unknown^22^. Animal studies have shown that IL-4 increased the survival of mice exposed to lethal doses of LPS4, and anti-inflammatory activity in several autoimmune diseases^16^. In this research, the surgical control group had IL-4 levels higher than those with sepsis, but no references were found that could explain this fact. This probably contributed to attenuate lesions in the pulmonary alveoli, classifying it as a protein with potential for the treatment of sepsis. However, as this study used a single dose for treatment, we suggest new ones addressing different concentrations and dosages to accurately identify all effects of Hev b 13 on sepsis and its mechanisms of action.

## CONCLUSION

The Hev b 13 fraction presents an anti-inflammatory tendency with potentials in the treatment of sepsis.
